# Prevention of stroke and cognitive decline in pediatric population in resource-limited settings

**DOI:** 10.3389/fstro.2024.1390220

**Published:** 2024-11-08

**Authors:** Ukamaka Dorothy Itanyi, Obiageli Eunice Nnodu

**Affiliations:** ^1^Department of Radiology, College of Health Science University of Abuja, Gwagwalada, Nigeria; ^2^Department of Haematology and Blood Transfusion College of Health Sciences University of Abuja, Gwagwalada, Nigeria

**Keywords:** stroke, pediatric, prevention, resource-limited setting, cognitive decline

## Abstract

There is an increasing global burden of pediatric stroke especially in low- and middle-income countries (LMICs). This is worsened by the specific risk factors in these areas, including Sickle Cell Disease and endemic infections like Tuberculosis and Human Immunodeficiency disease. Stroke occurs 221–300 times more frequently in patients with SCD when compared to healthy children. Although established stroke units and acute stroke care can improve outcomes, these are often not available in resource-poor settings. Primary and secondary prevention of strokes become a very important strategy to reduce the mortality and debilitating physical and cognitive long-term effects of stroke. There are myriads of challenges with implementing already established global policies and guidelines for stroke care in LMICs. These include paucity of data on this subject, poor knowledge and awareness about the symptoms of childhood stroke, adverse cultural beliefs regarding strokes, lack of screening and diagnostic equipment, inadequately trained manpower as well as nonexistent evidence-based management guidelines in these regions. To address these challenges, simple, cost-effective, stroke care models that determine the process of care and how available services should be delivered have been proposed to suit the peculiarities of LMICs in the areas of stroke risk assessment, prevention, and management.

## 1 Introduction

Despite its seeming rarity, there has been an increased global awareness and recognition of stroke as a significant cause of morbidity and mortality in children. There is however limited data on childhood stroke globally since it is generally underrecognized. This is worse in low, medium income countries (LMICs) with no significant data from Africa in the International Pediatric Stroke Study, IPSS (Bejot et al., 2008). This childhood stroke registry includes more than 100 institutions in 34 countries with over 8,000 children (Bejot et al., 2008). This under-representation and poor recognition of stroke in children is mainly due to poor awareness of the clinical signs and symptoms by healthcare providers in the emergency as well as the public (Ndondo and Hammond, 2022). Although less common than adult stroke, the associated long-term neurological deficits are equally devasting with correlated increases in health care costs. Since stroke can be prevented and treated in some patients, proper identification and determination of the etiology and risk factors becomes necessary.

## 2 Definitions, etiology, and risk factors

Stroke is defined as the sudden onset of focal, neurologic deficit persisting ≥24 h or until death, resulting from irreversible damage to the brain parenchyma following a cerebrovascular insult (Sacco et al., 2013). The vascular insult could lead to infarction, intra-cerebral hemorrhage, and/or subarachnoid hemorrhage (Sacco et al., 2013). Ischemic (infarction) stroke includes arterial ischemic stroke (AIS) and cerebral venous thrombosis with venous infarction while Hemorrhagic stroke (HS) includes intracerebral hematoma or subarachnoid hemorrhage (Jordan, 2008).

### 2.1 Arterial ischemic stroke

Pediatric arterial ischemic stroke is defined as the onset of an acute neurological deficit with accompanying radiographic evidence of infarction in the corresponding arterial territory occurring in a child between 29 days and 18 years (Bernard et al., 2012). Arteriopathies, which are recognized as the most significant risk for AIS and the strongest risk of stroke recurrence in children, could be chronic or transient and are frequently detected on vascular imaging (Bohmer et al., 2019; Fullerton et al., 2016).

### 2.2 Hemorrhagic stroke, HS

This occurs in 50% of childhood strokes (Fullerton et al., 2016) and includes spontaneous intraparenchymal and non-traumatic subarachnoid hemorrhage. The commonest causative lesion is arteriovenous malformations (AVMs) in 30% with the probability of having a first hemorrhage occurring at 2–4% per year (Mallick and O'Callaghan, 2010).

### 2.3 Risk factors of pediatric stoke in LMIC

The etiologies and risk factors of pediatric stroke differ from those in adult stroke (Bernard et al., 2012). A large study by the IPSS involving 493 children with stroke reported that the risk factors are influenced by the age at presentation, geographical location, ethnicity, as well as available medical resources (Mackay et al., 2011).

Sickle cell disease (SCD) and endemic infectious diseases constitute significant risk factors for pediatric stroke in LMICs (Ndondo and Hammond, 2022). Studies done in India (Patra et al., 2015) and Pakistan (Siddiqui et al., 2006) reported intracranial infections as the commonest etiology of stroke, while some West African studies have reported SCD as the commonest risk factor for pediatric stroke worse with sickle cell anemia which is the homozygous form (Ndiaye et al., 2018; Fatunde et al., 2005). A study done in Kenya, East Africa however reported connective tissue disease and congenital heart disease as the commonest risk factors for AIS (Ogeng'o et al., 2010).

#### 2.3.1 SCD vasculopathy

Hematological disorders, commonly SCD in African children, markedly increase the risk of stroke, with up to a third of patients having neuroimaging abnormalities without a history of overt stroke (Ciceri et al., 2011). The mechanism of stroke in SCD is likely a result of background chronic anemia and the hypercoagulable state as well as stenosis of the distal internal carotid artery (ICA) and/or the proximal middle cerebral artery (MCA) secondary to chronic repetitive insults on the vasculature (Ciceri et al., 2011; Idro et al., 2022; Kija et al., 2019; Elmahdi et al., 2022; Jacob et al., 2020).

Another form of arteriopathy in SCD is Moya Moya which is a non-inflammatory vasculopathy characterized by progressive stenosis of vessels feeding the circle of Willis with a compensatory network of collateral vessels distally (Ciceri et al., 2011). This is seen on angiograms as a “puff of smoke” and is seen in a proportion of patients with SCD and stroke (Idro et al., 2022; Kija et al., 2019; Elmahdi et al., 2022; Jacob et al., 2020). The risk of stroke recurrence has been reported to be significantly higher for those who have moyamoya collaterals (Dobson et al., 2002).

Silent cerebral infarct (SCI), the most common form of neurologic disease in pediatric SCD, is defined as abnormal magnetic resonance imaging (MRI) findings without an overt clinical neurological deficit. It is noted as an independent risk factor for overt stroke and occurs in up to 37% of these children by the 14th birthday (DeBaun et al., 2012). A prospective study of 224 children with Sickle cell anemia in Tanzania reported a prevalence of 27% in neurologically asymptomatic children (Jacob et al., 2020).

SCD is also a major risk factor for hemorrhagic stroke in children occurring in up to 25% of first strokes in this subset of children (Ciceri et al., 2011). The Baltimore-Washington Cooperative Young Stroke Study reported a nearly 250-fold risk of HS in children with SCD compared with unaffected children (Earley et al., 1998). HS is more likely in children who have had a previous ischemic stroke, acute hypertension, a low steady-state hemoglobin, leukocytosis, acute chest syndrome, and a history of hypertransfusion or have received steroids (Strouse et al., 2006; Ohene-Frempong et al., 1998).

#### 2.3.2 Endemic infections

The pathophysiology of encephalitis and meningitis as risk factors is presumably due to associated inflammation of the vasculature, reduction in perfusion due to accompanying hypotension, thrombosis, reduction in cerebrospinal fluid glucose as well as intracranial hypertension (Ciceri et al., 2011). Tuberculous meningitis (TBM) is endemic in LMIC. The affected children have been noted to have an increased stroke risk with a large percentage presenting initially with stroke at admission and a significant number at the end of the first month of presentation (Solomons et al., 2021).

Bacterial meningitis, which is an important stroke risk and a major cause of morbidity and mortality in children, causes vasculopathy in the region of the infective focus and is associated with ischemic stroke in 10–25% of cases (Ndondo and Hammond, 2022). Pneumococcal meningitis and Haemophilus as well as Group B streptococcus in neonates are reported to cause more strokes in children than meningococcal meningitis (Pryde et al., 2013).

Human immunodeficiency virus (HIV) infection is equally common and is also recognized as a stroke risk in LMICs with both ischemic and hemorrhagic stroke occurring in infected persons (Cole et al., 2004; Qureshi et al., 1997). Although clinical series reported that between 1.3 and 2.6% of children infected with HIV develop stroke (Park et al., 1990; Patsalides et al., 2002), the extent of cerebrovascular disease is thought to be underestimated in LMICs due to limited access to neuroimaging (Kolapo and Vento, 2011). Stroke in HIV patients has been independently associated with arteriopathy of the cerebral vessels, especially in younger patients, and can be the first presentation of HIV infection in children (Visudtibhan et al., 1999). A study comparing people living with HIV (PLWH) and HIV-negative patients presenting with acute stroke at a large tertiary hospital in Cape Town, South Africa, reported that PLWH were nearly a decade younger, had less traditional risk factors for stroke, and were more likely to have a concurrent infection with CD4 counts of < 200 cells/μL (Corbett et al., 2022). Ischemic strokes in this group were also more likely to affect more than one vascular territory. Besides the virus, opportunistic infections, coagulopathies, and the antiretroviral therapy instituted for treatment are reported to increase stroke risk further (Cole et al., 2004; Qureshi et al., 1997).

Rheumatic heart disease (RHD), secondary to Streptococcus pyogenes infection is reported as the most common form of cardiac pathology in people of low socioeconomic status with an estimate of 3–7.5% of all strokes in LMICs related to RHD (Carapetis et al., 2005).

Childhood stroke in which no risk factors are identified represents 10–30% of cases (Mallick and O'Callaghan, 2010).

## 3 Prevalence of stroke in children in LMICS

There is a paucity of data on the incidence and prevalence rates, types and etiology, associated risk factors, morbidity, and mortality of stroke across the lifespan of children in LMICs (Ndondo and Hammond, 2022) mostly as a result of lack of clinical suspicion. Analysis of the sociodemographic differences in prevalence, incidence, mortality, and disability-adjusted life-years (DALYs) of stroke among children aged 0–14 years using the 2019 Global Burden of Disease Study, reported that incident strokes and prevalent strokes increased by 18.51 and 31.97% globally (Du et al., 2023). They reported a disproportionate burden in LMICs which had 84% of incident cases, 83% of prevalent cases, 80% of years lost due to disability, and 93% of years of life lost in 2019, surpassing the global average. Eastern, Southern, and Western sub-Saharan Africa were identified as the regions that had the highest incidence of stroke primarily due to the high burden of SCD (Du et al., 2023).

More studies of prevalence rates have however been done around SCD and stroke since this is the most common risk factor for stroke in black children with more than 75% of the global burden of SCA occurring in sub-Saharan Africa (Piel et al., 2013). Stroke is 221–300 times more frequent in patients with SCD when compared to healthy children with the greatest incidence between 4 and 15 years of age (Ohene-Frempong et al., 1998). The first systematic review that analyzed neurologic events in African children with SCD reported a stroke prevalence of 4.2% in a pooled sample of 18,977 children obtained from 23 studies (Noubiap et al., 2017). Nigeria has the largest population of SCD and consequently a high burden of stroke with reported pediatric stroke prevalence ranging from 2.9 to 8.6%. Other LMICs have prevalence rates that are comparable to this (Njamnshi et al., 2006; Babela et al., 2005; Lagunju and Brown, 2012; Madu et al., 2014; Jiya et al., 2015; Munube et al., 2016; Amayo et al., 1992; Heimlich et al., 2016; Saidi et al., 2016; Adegoke et al., 2015) (see Table 1).

**Table 1:** Prevalence rate of stroke in Pediatric SCD diseases in some LMICs.

**Country**	**Study period**	**Age range (years)**	**Sample size**	**Stroke prevalence**	**References**
Cameroon	Oct–Dec 2002	0.7–35	120	6.67%	Njamnshi et al., 2006
Congo	May 1995–May 2002	0.5–17	1,422	1.1%	Babela et al., 2005
SW, Nigeria	July 2005–June 2011	1–16	214	8.4%	Lagunju and Brown, 2012
Nigeria (Multicentre)	June 2012–June 2014	< 18	2,955	7.4%	Madu et al., 2014
NE, Nigeria	May 2004–Apr 2014	0–15	416	3.6%	Jiya et al., 2015
Uganda	Aug 2012–Aug 2014	< 18	2,810	6.8%	Munube et al., 2016
Kenya	Jan 1983–Jan 1988	0.6–21	360	3.3%	Amayo et al., 1992
Malawi	May 2018	2–10	117	8.5%	Heimlich et al., 2016
Tanzania	Aug–Sep 2012	1–15	124	16.9%	Saidi et al., 2016
Nigeria	Jan–Dec 2013	0.5–15	240	2.9%	Adegoke et al., 2015

## 4 Clinical manifestations

The clinical evaluation of pediatric stroke is more challenging compared with adults. This is partly a result of poor clinical suspicion due to atypical, varied, and non-pathognomonic clinical presentations leading to delayed diagnosis (Hollist et al., 2021). Although the recognition and evaluation of pediatric stroke is as urgent as that in adults with established protocols for both age strata, recognition of stroke symptoms in children still poses a challenge. Acute hemiparesis, which is recognized as the hallmark sign of stroke particularly in adults, can be seen in older children while seizures are the predominant presentation in younger children (deVeber et al., 2017). Children under 1 year are more likely to present with seizures and encephalopathy than focal signs (Ndondo and Hammond, 2022). Other non-specific symptoms such as headache, fever, nausea, and vomiting as well as cardio-pulmonary dysfunction can also be seen. A study in India reported hemiparesis in 70.6% of children followed by seizure, altered consciousness, both fever and cranial nerve palsy, vomiting, and aphasia in decreasing frequency (Patra et al., 2015). A similar study in Senegal reported predominantly hemiplegia (33.33%) and signs of raised intracranial pressure (20.51%) in children with hemorrhagic strokes while hemiplegia (79.60%) and seizures (18.05%) were predominant in children with ischemic stroke (Ndiaye et al., 2018). The confusion in diagnosis is further worsened by stroke mimics such as migraines, intracranial tumors, focal seizures, and demyelinating diseases which can cause acute-onset focal neurologic symptoms in 21–76% of affected children (Lehman et al., 2019). Given the associated neurologic and cognitive deficit and urgent treatment requirements, it is recommended that stroke should be considered as a differential diagnosis for acute-onset neurologic deficit in children, especially in the presence of risk factors (Ndondo and Hammond, 2022).

## 5 Diagnosis

The pathways for diagnosis and care for pediatric stroke are not as structured as the adult system. However, like adult stroke, neuroimaging is vital in confirming pediatric stroke and ruling out mimics. The Australian Childhood Stroke Advisory Committee recommends that stroke must be confirmed on imaging before instituting reperfusion treatment (Medley et al., 2019). Cranial MRI and angiography are recognized as the neuroimaging modalities of choice (Medley et al., 2019). The MRI sequences are especially important in differentiating between stroke and mimics. MRI is non-ionizing with an excellent spatial resolution, however, the non-availability especially in LMICs, the exorbitant cost of the few machines, and the long duration of image acquisition requiring sedation, as well as contraindications in patients with ferromagnetic implants are disadvantages to its use. It is estimated that the MRI density per million populations in LMICs is 0.04 in Cameroon to 1.33 in Bhutan (Tan et al., 2022). Cranial computed tomography, though ionizing, is recommended as a viable alternative if MRI is not available. The ready availability in emergency departments, non-necessity of sedation in most patients, fast acquisition of images, and high accuracy in the detection of hemorrhage are advantages of CT. CT angiography can further identify other features that are associated with AIS such as vascular occlusion and arterial dissection. However, there is evidence of poor sensitivity of CT in detecting hyperacute ischemic infarction leading to further delayed diagnosis (Medley et al., 2019).

## 6 Sequalae of pediatric stroke

Stroke is one of the top ten causes of death in children with long-term neurological sequelae noted in more than half of the survivors (Ciceri et al., 2011). Between 50 and 80% of survivors are reported to have permanent neurological deficits, especially hemiparesis or hemiplegia (Ellis et al., 2014). Pediatric stroke has been recognized to lead to multiple debilitating disorders including sensorimotor deficits, behavioral issues, intellectual disability, language impairment, and epileptic seizures (Felling et al., 2017) which are reported to occur in ~15–20% of children with childhood AIS and ≤ 17% of children with hemorrhagic stroke (Sacco et al., 2013). Children whose initial presentations were altered consciousness, infarcts bilaterally, and imaging evidence of arteriopathies are reported to be more likely to have long-term neurologic deficits (Greenham et al., 2016).

### 6.1 Cognitive impairment

Cognitive impairments, which are present in about 20–50% of the children, affect both behavioral traits and executive functions (Mackay and Steinlin, 2019). Studies concerning the relationship between the age of the children at initial stroke and cognitive outcome have differed over the years. It is, however, recognized that the changes in cognition can start and increase over time with the full sequelae emerging many years after the acute stroke event (Westmacott et al., 2009). A recent study investigating the effect of age at pediatric AIS on long-term cognitive outcome reported that a stroke in early childhood (29 days to < 6 years) was associated with a significantly worse outcome for cognitive flexibility, processing speed, and verbal learning irrespective of the size and location of the lesion (Abgottspon et al., 2022). Neonatal and later pediatric strokes were noted to have better cognitive outcomes.

A recent cross-sectional study evaluating neurocognitive impairment in Ugandan children with sickle cell anemia when compared to sibling controls revealed poorer cognitive and executive function in children with sickle cell anemia (SCA) aged between 5 and 12 years and those with prior stroke (Bangirana et al., 2024). Similarly, a Nigerian study reported that transcranial Doppler (TCD) velocity is significantly related to executive function efficiency in children with SCA (Mboizi et al., 2024). A Ugandan study assessing the neurological and cognitive impairment in children with SCA (BRAIN SAFE) noted that >20% of the children had ≥1 impairment (Green et al., 2019).

Silent cerebral infarction has been associated with cognitive impairment (Jacob et al., 2020).

A meta-analysis by Kawadler et al. (2016) on the effect of SCI on full-scale IQ (FSIQ) found that children with SCA and SCI scored significantly lower than children with SCA without SCI. Other Studies have also shown that children with SCD and SCIs performed worse on tests of vocabulary, coordination, visual-motor speed, and arithmetic when compared to children with SCD who had abnormalities on imaging evaluation (Armstrong et al., 1996; Wang et al., 2001).

HIV-associated vasculopathy is recognized to be associated with cognitive impairment. The clinical features may be subtle or silent or consist of neurobehavioral issues, learning difficulties, or regression (Izbudak et al., 2013).

Adequate post-stroke care and rehabilitation are hampered globally by fragmentation and poor coordination of care, a situation worsened by the added challenges of poor access to specialized stroke care services as well as the lack of adequately trained personnel in LMICs (Mboizi et al., 2024). Continuity of stroke rehabilitation post-discharge is therefore a major challenge in these areas.

## 7 Prevention of stroke and cognitive decline

Unlike adult stroke, the diverse nature of the etiology and risk factors of pediatric stroke makes prevention rather difficult to achieve. Following the paucity of dedicated stroke units, imaging modalities, and adequate therapeutic measures that ensure better patient outcomes in low-resource regions, there is a need for adequate primary and secondary preventative measures as important strategies for the reduction of mortality and disability in preventable cases of stroke. Recognition and screening for peculiar risk factors are useful for the overall prevention of stroke in adults as well as children.

### 7.1 Primary stroke prevention in children with sickle cell disease

SCD has been recognized as the only pediatric stroke risk factor that has evidence-based management which is anchored on randomized control trials (Adams et al., 1998).

#### 7.1.1 Chronic blood transfusion

The Stroke Prevention Trial in Sickle Cell Anemia (STOP) established that chronic blood transfusion (CBT) therapy, can reduce the risk of a first stroke by 92% in high-risk children with abnormal cerebral blood flow velocities noted on transcranial Doppler (TCD) ultrasound (Adams et al., 1998). Following the successes recorded, the American Society of Hematology (ASH) guideline panel strongly recommended annual TCD screening for the prevention of stroke in this subset of children. Those with abnormal TCD velocities are recommended to have regular blood transfusion therapy, typically every 3–4 weeks for at least a year, to maintain the maximum HbS level < 30% and maintain the hemoglobin level >9.0 g/dL (DeBaun et al., 2012). Despite the remarkable stroke prevalence reduction, the challenge of excessive Iron storage following repeated blood transfusion made the use of monthly blood transfusion burdensome. Notwithstanding the documented success, the use of chronic blood transfusion is not feasible in poor resource settings. Lagunju et al. (2013) in a study done in Nigeria, reported a low acceptance rate of chronic transfusion therapy among caregivers of children with SCD largely due to high economic costs, difficulties with finding donors, and unavailability of blood. This is further worsened by cultural beliefs attributing stroke to diabolic forces, as well as poverty all leading to poor uptake and compliance with CBT among patients and their families.

#### 7.1.2 Hydroxyurea

These challenges further informed a phase III randomized controlled trial, TCD with Transfusions Changing to Hydroxyurea (TWiTCH). This demonstrated the effectiveness of Hydroxyurea (HU), a myelosuppressive, chemotherapeutic agent, in maintaining TCD velocities and preventing primary stroke in children with abnormal CBFV after an initial year of blood transfusion if there is no evidence of severe vasculopathy on MRA (Ware et al., 2016).

To combat the challenges, the Stroke Prevention in Nigeria (SPIN NCT01801423) feasibility trial conducted in 2012 to determine the uptake and effectiveness of HU therapy for primary stroke prevention in children with abnormal TCD velocities showed an appreciable reduction in CBF velocities within 3 months by ~85% (Galadanci et al., 2017). They also noted stroke risk reduction at a dose of 20 mg/kg/day as well as a high rate of participant recruitment, retention, and adherence for HU. A later study by Lagunju et al. (2019) done in Nigerian children with SCD and abnormal CBF velocities also reported a significant reduction in TCD velocities and a corresponding reduction in the incidence of primary stroke following treatment with HU. Following a mean follow-up of 3.6 years, only one stroke event occurred in the cohort, giving a stroke incidence of 0.27/100 person-years. Another multicenter Trial conducted in Northern Nigeria (SPRING, Primary Stroke Prevention in Nigeria, NCT02560935) concluded that a fixed low-dose HU of at least 10 mg/kg/day was effective for the prevention of primary stroke (Abdullahi et al., 2022). Further analyses however suggested that the moderate-dose group (20 mg/kg/day) was less likely to be hospitalized for all other causes.

### 7.2 Secondary stroke prevention in SCD

The current recommendations secondary stroke prevention and treatment children with SCD who presented with signs and symptoms of stroke include a simple blood transfusion top-up urgently done within 2 h to increase oxygen delivery if hemoglobin is < 10 g/dl. Furthermore, exchange blood transfusion is suggested to reduce the sickled hemoglobin to < 30% while also aiming to increase the hemoglobin concentration to >10–11 g/dL (DeBaun et al., 2012). Further research reported that following a prior overt stroke and monthly blood therapy, there was still a 45% chance of infarct recurrence within 5.5 years while still on chronic blood transfusion (Hulbert et al., 2011). However, there was a strong association between progressive cerebral vasculopathy and infarct recurrence, relative risk 12.7 (95% CI 2.65–60.5, *P* = 0.001) (Hulbert et al., 2011).

An earlier study by Lagunju et al. (2013) in Nigeria reported that the risk of recurrence and severe motor disability was reduced in children with a first clinical stroke following the use of Hydroxyurea. This finding was further corroborated by the SPRINT (hydroxyurea for secondary stroke prevention in children with sickle cell anemia in Nigeria) trial, a randomized, double-blind trial of 2 fixed-dose regimens of hydroxyurea, which provided evidence that both low- and moderate-dose hydroxyurea regimens are effective for secondary stroke prevention when chronic transfusion therapy is not feasible (Abdullahi et al., 2023).

For children with Moyamoya and stroke or transient ischemic attack, the American Society of Hematology guideline suggests evaluation for revascularization surgery and regular blood transfusion as the secondary strategy to prevent stroke recurrence (DeBaun et al., 2012).

### 7.3 Silent strokes and cognitive function

Given the high prevalence of silent cerebral infarcts (silent stroke) in children with SCD and their association with cognitive impairment, the American Society of Hematology (ASH) guidelines recommend at least one non-sedated screening MRI in early school-age children with SCA to detect silent infarcts and surveillance MRI of the brain every 1–2 years to identify disease progression (DeBaun et al., 2012). On identification of silent infarct, a formal expert cognitive assessment is recommended to secure additional resources for the rehabilitation and institution of special educational services for the child. Further recommendations include the involvement of family and caregivers on the need for regular blood transfusion therapy for at least 3 years to prevent further recurrence and neurological damage (DeBaun et al., 2012).

Following the recognition of cognitive impairment in Ugandan children with SCD, a further open-label trial (BRAIN SAFE II) showed significant improvement in attention and cognition following therapy with HU suggesting its role in enhancing overall cognitive function in this subset of children. Similarly, findings of decreased TCD velocity and improved cognition with early use of hydroxyurea have been reported by other authors (Wang et al., 2021; Heitzer et al., 2021).

### 7.4 Other primary preventative strategies for pediatric stroke

The study by the International Pediatric Stroke Study registry reported infection as a significant risk factor for stroke in 24% of 676 AIS cases enrolled in 10 countries (Bejot et al., 2008). Vaccination against infectious diseases that constitute risk factors, as well as early diagnosis and adequate treatment of the endemic pathogens will also form a basis for the prevention of pediatric stroke. The Expanded Programme on Immunization (EPI) program was created in 1974 by the World Health Organization (WHO, 1974) to improve the availability of vaccines globally. Vaccination with Bacille Calmette-Guerín (BCG) was incorporated into childhood immunization programs in countries with high TB disease burdens. WHO strongly recommends that BCG vaccination which is most effective against TB and TBM should be given to all neonates in settings with a high tuberculosis burden (Feikin et al., 2006). The development and introduction of low-cost vaccines into routine immunization programs in high-burden countries has led to a significant reduction in meningitis infection and consequent neurological effects (Alderson et al., 2021). Likewise, the progression of throat infection by Group A Streptococcus, GAS leading to acute rheumatic fever (ARF) and rheumatic heart disease (RHD) in children can be curbed by primary and secondary preventive measures using cost-effective Benzathine penicillin (BPG) (Manyemba and Mayosi, 2002). Furthermore, Echocardiography-based screening has been used to detect latent cardiac diseases that can be treated successfully (Ali and Subahi, 2020).

With regards to HIV- associated vasculopathy, optimization of HIV virological control, exclusion of opportunistic infections, and the avoidance of ART with adverse atherosclerotic side effects have been recommended as the management focus in this subset of patients (Hammond et al., 2016).

## 8 Lessons learned from studies in resource-limited settings

The implementation of several strategies has led to gains recorded in the prevention and care of pediatric stroke in some resource-limited regions.

### 8.1 SCD

With regards to SCD, a recent update of the evidence in LMICS for the management of pediatric SCD by Odame (2023) recommended strategies for early diagnosis through newborn Screening (NBS) programs. Easy cost-effective point of care assessment has been developed and used successfully by the ASH. An observational study in Haiti using the rapid point-of-care (POC) Sickle SCAN revealed excellent specificity and sensitivity to detect SCD during newborn screening and shortened healthcare access for children positive for SCD (Alvarez et al., 2019). Early detection of SCD further allows for better screening and primary prevention of stroke in these children.

Furthermore, one of the goals of the SPIN trial in Nigeria included having a sustainable stroke prevention program. They successfully constituted a Sickle Cell Disease Stroke Prevention Team, the first regional program in Africa that recorded a 100% maintenance and implementation rate after 1 year, further demonstrating the feasibility and sustainability of pediatric stroke prevention in Africa (Ghafuri et al., 2022).

In addition, HOPE-KIDS 2 (NCT04218084), a phase 3, multicenter, placebo-controlled trial carried out between 2020 and 2023 to evaluate the effect of Voxelotor (a hemoglobin S polymerization inhibitor) treatment on reducing stroke risk in children with SCD concluded that the clinical sites (Africa and Middle Eastern sites) that were studied successfully implemented a standardized, local TCD screening protocol with relatively few limitations regarding participant screening (Bello-Manga et al., 2023).

### 8.2 Endemic infections

TBM is recognized as one of the leading causes of neurological morbidity and mortality in LMICs, the development of affordable, accurate diagnostic testing for TBM remains a priority as well as the availability of affordable, safe, and effective anti-tuberculous drugs (Ndondo and Hammond, 2022).

The Global Roadmap to Defeat Meningitis by 2030 is an initiative that seeks to raise awareness of bacterial meningitis and to reverse the trend by emphasizing prevention through adequate vaccination. The development, availability, and use of low-cost vaccines to prevent Hemophilus influenzae type B, Hib and pneumococcus have significantly impacted the prevalence of meningitis and other disease manifestations caused by these pathogens (Alderson et al., 2021). Currently, Nigeria has become the first country to receive the new MenFive meningitis vaccine which targets five main strains of meningococcal meningitis prevalent in Africa from the Vaccine Alliance-funded global stockpile^1^.

With regards to HIV infection, which is a notable risk factor for pediatric stroke, there has been a progressive decline in the rate of new infections among children since the introduction in 2011 of the “Global Plan towards the Elimination of New HIV Infections among Children and Keeping their Mothers Alive”. This is mainly because of increased access to prevention of mother-to-child transmission (PMTCT) related services and an increased number of pregnant women living with HIV being initiated on lifelong antiretroviral medicines with Eastern and Southern Africa showing sustained progress in PMTCT of HIV (UNICEF).

To address problems of control of RHD, the SUR I CAAN (Surveillance, Integration, Communication, Awareness, Advocacy, and Training) program was implemented in Sudan with the aim of improving primary, secondary, and tertiary prevention of RHD. They instituted the use of point-of-care handheld echocardiography for early detection and treatment of RHD (Ali and Subahi, 2020). Analysis done in Brazil showed that echocardiographic screening utilizing handheld devices, performed by non-physicians with remote interpretation by telemedicine is cost-effective (Ubels et al., 2020).

### 8.3 Limitations of primary stroke prevention

Although several strategies have been implemented and gains recorded in the prevention and care of pediatric stroke, there are still myriads of challenges encountered in addressing the stroke burden among children in LMICs. These include a paucity of relevant data for policymaking, as well as limitations at the patient and hospital levels (Ndondo and Hammond, 2022).

Odame (2023) reported a high SCD disease burden with poor outcomes which are worsened by inadequate healthcare infrastructure, and endemic infectious comorbidities like malaria and HIV. Bello-Manga et al. (2022) in a study on SCD in Northern Nigeria, reported that only 5.49% of eligible children ever had TCD screening in their lives, mainly due to a lack of trained personnel and machines.

Despite successes recorded in tackling infectious diseases through vaccination, vaccine skepticism and hesitancy have increased in LMICs, a situation worsened by the COVID-19 pandemic and the associated misinformation concerning the safety of vaccines in general (Simas and Larson, 2021).

Other limitations have been noted in the areas of pediatric stroke care. At the patient level, there is a delay in assessing care for children with neurologic signs and symptoms largely due to a lack of knowledge concerning the signs and symptoms of stroke, in children. Studies from India (Ashraf et al., 2015) and Nigeria (Philip-Ephraim et al., 2015) indicate lengthy delays in presenting to the hospital, with < 30% of patients with stroke attending the hospital within 3 h of symptom onset.

Further barriers noted within the hospital include a lack of acute stroke care protocol and units, unavailability of brain imaging equipment as well as a dearth of specialists like neurologists, radiologists, physiotherapists, occupational therapists, and nurses. Although critical to the diagnosis of stroke, Africa records very poor access to neuroimaging services (Tan et al., 2022). Cerebrovascular disease has been reported to be frequently undetectable by clinical assessments alone and therefore the need for neuroimaging in at-risk children (Idro et al., 2022).

### 8.4. Proposed solutions to combat LMIC-specific challenges

To combat the challenges of primary stroke prevention in children with SCD, Bello-Manga et al. (2022) proposed an alternate “task shifting” implementation strategy focused mainly on training nurses within the community health centers on skills of TCD screening as well as training the non-specialist medical officers within these establishments on the initiation and monitoring of HU administration to children with abnormal TCD measurements.

Appropriate vaccination, early diagnosis, and treatment of endemic infectious constituting stroke risks would reduce the burden of stroke in childhood. There is an urgent need to make safe and effective anti-tuberculous and anti-retroviral drugs (with the availability of combination formulations) widely available (Ndondo and Hammond, 2022).

To combat difficulties in clinical diagnosis and symptom recognition, the 2017 guidelines of the Royal College of Pediatrics suggest increasing awareness of stroke in childhood amongst the public and front-line clinical staff, by emphasizing the use of the FAST (face, arm, speech, time) tool in the pediatric population (RCPCH, 2017). Lately, the Questionnaire for Verifying Stroke-Free Status (QVSFS), an 8-item structured questionnaire that is a simple, accurate, and cost-effective diagnostic screening tool was adopted in Ghana to address the challenges of screening and detection of strokes. The validated version of the questionnaire which would also include an app, will be used to screen all SCD patients in registries in Ghana and Nigeria during routine follow-up to capture stroke events clinically followed by detailed evaluation (Arfo et al., 2016).

Given the several limitations, alternative stroke models of care have been proposed to suit the peculiarities of LMICs. These models include (i) Multidisciplinary team care, (ii) Specialist-led care, (iii) physician-led care, (iv) hub and spoke model, and (v) task sharing (Pandian et al., 2020). Though used in adults, they can be adapted for the purpose of early diagnosis and treatment of acute pediatric stroke. The task-sharing model has been suggested in Nigeria given the dearth of trained personnel (Alderson et al., 2021). A study done to assess the effect of implementing stroke unit care in a rural Indian hospital using the physician-led care model showed improvements in quality measures, complication rates, and patient outcomes (John et al., 2021).

Furthermore, few cost-effective and alternative models of care have been proffered by Pandian et al. (2017) for establishing feasible stroke care services. These include the generation of stroke awareness in the lay public as well as addressing cultural beliefs that might delay rapid treatment, incorporation of multidisciplinary team members into ward rounds, identifying discharge needs and recovery goals of the patient, and training of primary caregivers about rehabilitation.

For stroke care to be effective in low-resource settings, there is a need to define the vital areas of care that will be of greatest benefit using the available resources. Four different components have equally been defined as requirements to improve stroke care systems in LMICs (Simas and Larson, 2021). These include (i) collection of high-quality hospital and population-level data on stroke, (ii) identification of elements of organized stroke care that can be implemented to make the largest gain when there is no access to costly high resources, (iii) specialized multidisciplinary stroke training using optimal evidence-based care for the unique setting, and (iv) integration of stroke services into national non-communicable diseases programs to achieve the UN Sustainable Development Goal 3.4 (Pandian et al., 2017).

## 9 Conclusions

The challenges in pediatric stroke care in LMIC countries are myriad and are worsened by the peculiarity of sickle cell disease and some endemic infections as LMIC-specific risk factors for stroke. The use of TCD for screening stroke risk in children with sickle cell disease and the primary prevention of stroke with hydroxyurea are proven strategies for stroke prevention for this subset of children, likewise the use of vaccinations for endemic infections.

Furthermore, several models of care involving local and national governments for stroke care have been established in low-resource settings to improve outcomes for stroke patients. The recent expansion in the field of stroke care in low-resource regions calls for more integrated efforts at personal, hospital, community, national, and international levels for more positive and fruitful collaborations. The implementation of the different strategies will undoubtedly reduce the burden of stroke incidence in Africa and consequently mortality and morbidity as well as the associated neurocognitive sequelae.

## Footnotes

^1^Available at: https://www.healthnews.ng/nigeria-leads-the-way-with-new-vaccine-to-tackle-meningitis-outbreak/ (accessed April 22, 2024).
